# Maternal impulse control disability and developmental disorder traits are risk factors for child maltreatment

**DOI:** 10.1038/s41598-017-14666-5

**Published:** 2017-11-14

**Authors:** Yoshiyuki Tachibana, Kenji Takehara, Naoko Kakee, Masashi Mikami, Eisuke Inoue, Rintaro Mori, Erika Ota, Tomoe Koizumi, Makiko Okuyama, Takahiko Kubo

**Affiliations:** 1 Division of Infant and Toddler Mental Health, Department of Psychosocial Medicine, National Centre for Child Health and Development, Tokyo, Japan; 2 Department of Health Policy, National Research Institute for Child Health and Development, Tokyo, Japan; 3 Division of Bioethics, National Centre for Child Health and Development, Tokyo, Japan; 4 Department of Biostatistics, Clinical Research Centre, National Centre for Child Health and Development, Tokyo, Japan; 5 National Research Institute for Child Health and Development, Tokyo, Japan; 6 Department of Psychosocial Medicine, National Centre for Child Health and Development, Tokyo, Japan; 7 Department of Perinatal Medicine and Maternal Care, National Center for Child Health and Development, Tokyo, Japan; 8 Present Address: Division of Medical Informatics, St. Mariann University School of Medicine, Kawasaki, Japan; 9 Present Address: Global Health Nursing, Graduate School of Nursing Science, St. Luke’s International University, Tokyo, Japan; 10 Present Address: Shirota Obstetrical and Gynecological Hospital, Zama, Japan

## Abstract

Previous work has suggested that maternal developmental disorder traits related to autism spectrum disorder (ASD) and attention-deficit hyperactivity disorder (ADHD) are significantly associated with child maltreatment. However, there may be other important maternal characteristics that contribute to child maltreatment. We hypothesized that maternal impulse control disability may also affect child maltreatment in addition to maternal developmental disorder traits. We aimed to test this hypothesis via a cohort study performed in Tokyo (n = 1,260). Linear regression analyses using the Behavioural Inhibition/Behavioural Activation Scales, the self-administered short version of the Pervasive Developmental Disorders Autism Society Japan Rating Scale, the short form of the Adult Attention-Deficit Hyperactivity Disorder Self-Report Scale, and the Child Maltreatment Scale, revealed that excessive inhibition of behaviour and affect, which is impulse control disability, is significantly associated with child maltreatment (b = 0.031, p = 0.018) in addition to maternal developmental disorder traits (ASD: b = 0.052, p = 0.004; ADHD: b = 0.178, p < 0.001). Logistic regression analyses revealed that ASD (adjusted odds ratio [AOR] = 1.083, p = 0.014) and high behavioural inhibition (AOR = 1.068, p = 0.016) were significantly associated with moderate child maltreatment, while ADHD was associated (AOR = 1.034, p = 0.022) with severe child maltreatment. These maternal characteristics may inform the best means for prevention and management of child maltreatment cases.

## Introduction

Maternal developmental disorders traits related to autism spectrum disorder (ASD) and attention-deficit hyperactivity disorder (ADHD) have been suggested to associate with child maltreatment^1^. However, the diagnostic criteria for ASD/ADHD^2^ do not include any psychological characteristics directly related to child maltreatment, and these psychological characteristics may sometimes occur in individuals with ASD/ADHD but are not classified as symptoms. Furthermore, other maternal psychological characteristics besides ASD/ADHD related to child maltreatment may exist.

Wiehe *et al*. demonstrated abusive parents had a higher tendency of impulse control disability, leading to child maltreatment^3^. One of the more prominent biological vulnerability models of impulse control disability derives from Gray’s behavioural inhibition system (BIS) and behavioural activation system (BAS)^4–7^. Gray proposed that BIS and BAS underlie behaviour and affect resulting in impulsivity^8,9^. Gray’s BIS/BAS model has been used for conceptualizing theories of impulsivity (e.g.^5–7^). Negative feelings such as fear, anxiety, and frustration associated with impulsivity have been suggested to be related to BIS^10–12^. Sensitivity to reward cues and initiation of behavioural approaches related to impulsivity have been suggested with BAS^8,12^. At an extreme, a heightened BAS sensitivity may implicate a sociopathic personality^6,13^. Carver developed the BIS/BAS Scales to measure impulsivity based on the BIS/BAS model^14^. Impulsivity measured by the BIS/BAS Scales is posited to serve as a correlate to psychopathologic impulse control disability responsible for child maltreatment. To the best of our knowledge, previous studies on maternal psychological characteristics related to child maltreatment have not addressed this matter from the perspective of both maternal impulse control disability and developmental disorder traits such as ASD/ADHD. We hypothesized that maternal impulse control disability is significantly associated with child maltreatment in addition to maternal developmental disorder traits. We investigated this hypothesis via conducting a cohort study on prenatal and postnatal mental health pathologies. Considering the effect of developmental disorder traits, we used the data from our cohort study to investigate how impulsive control disability in mothers, as measured by the BIS/BAS Scales, may affect child maltreatment.

## Results

**Figure 1:**
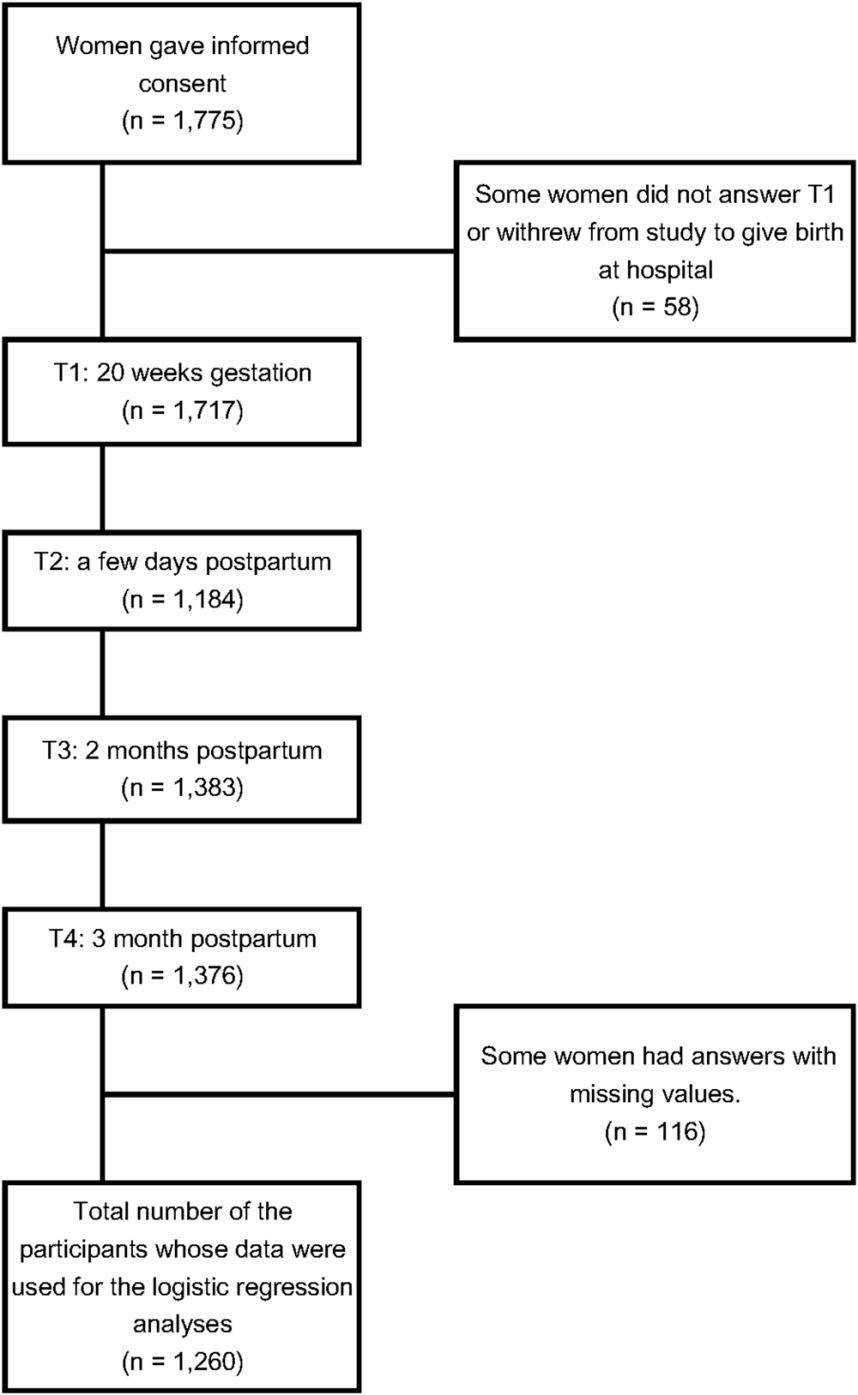
Flow chart of survey participation.

The recruitment process of study participants is shown in Figure 1. A total of 1,775 women who provided informed consent participated in this study. Among them, 1,717, 1,184, 1,383, and 1,376 of them answered the T1 (20 weeks gestation), T2 (a few days after delivery), T3 (two months after delivery), and T4 (three months after delivery) questionnaires, respectively. Table 1 shows the demographic, clinical, and socioeconomic characteristics of the participants. Data from 1,260 participants were included in the analysis, which were collected at T1, T2, and T3 (Table 1). The mean (±standard deviation [SD]) age of the participants was 35.05 (±4.38) years. Among them, 1,254 had partners and 2 did not (missing information from 4 participants). Regarding employment type, the number of full-time workers, part-time workers, temporary workers, and homemakers was 532 (42.22%), 153 (12.14%), 71 (5.63%), and 515 (40.87%), respectively. The number of participants with postgraduate, undergraduate, junior or technical college, high school, or junior high school education was 100 (7.94%), 664 (52.70%), 369 (29.29%), 118 (9.37%), and 9 (0.71%), respectively. The distribution of annual household income was 17 (1.35%) participants making <2 million yen, 250 (19.84%) making 2–4.9 million yen, 582 (46.19%) making 5–9.9 million yen, and 401 (31.83%) making >10 million yen (missing values from 13 participants). Regarding the number of deliveries, there were 895 (71.03%), 139 (11.03%), 44 (3.49%), 82 (6.51%), and 88 (6.98%) women who had had one, two, three, four, and five or more, respectively. One hundred sixty-one participants (12.78%) had a history of psychiatric treatment. The distribution of each item of the Child Maltreatment Scale (CMS)^15^ is shown in Table 2. The number of women whose total CMS scores were above the cut-off score for “at high risk of moderate child maltreatment” (2/3) and “at high risk of severe child maltreatment” (6/7) was 129 and 23, respectively. The means of the total scores of the CMS (±SDs) of all the participants, women at high risk of moderate child maltreatment, and women at high risk of severe child maltreatment were 1.20 (±1.68), 4.90 (±2.92), and 9.22 (±4.55), respectively. Table 3 shows the results of the cumulative ratio of the total CMS score. The ratios of the women whose total CMS scores were above the cut-off score for “at high risk of moderate child maltreatment” and “at high risk of severe child maltreatment” were 10.24% and 1.83%, respectively. Table 4 shows the multicollinearity of the linear regression analysis. The tolerance values and variance inflation factors (VIFs) in the models were less than 0.4 and 2.5, respectively, which revealed that there was no multicollinearity in Analyses 1, 2, and 3.

**Table 1:** Participants’ characteristics.

	Total				Moderate child maltreatment						p value	Severe child maltreatment					P value
	Number	Mean	SD		At high risk			Not at high risk				At high risk			Not at high risk		
					Mean	SD		Mean	SD			Mean	SD		Mean	SD	
		Range[Min, Max]			Range[Min, Max]			Range[Min, Max]				Range[Min, Max]			Range[Min, Max]		
Age (years)	1238	35.05	4.38	128	35.10	4.24	1110	35.05	4.39	23	0.89	33.43	4.69	1215	35.08	4.37	0.07
		[17,52]			[17,45]			[19,52]				[17,40]			[19,52]		
self-administered short version of the PARS score (ASD traits)	1260	1.03	1.24	129	4.39	3.28	1131	3.19	2.82	23	<0.001***	5.04	3.62	1237	3.28	2.87	<0.01**
		[0,6]			[0,17]			[0,18]				[0,14]			[0,18]		
ASRS score (ADHD traits)	1260	3.31	2.90	129	1.42	1.38	1131	0.99	1.21	23	<0.001***	1.91	1.41	1237	1.02	1.23	<0.001***
		[0,18]			[0,6]			[0,6]				[0,5]			[0,6]		
BIS score (Behaviour inhibition system)	1260	19.06	3.95	129	20.40	3.94	1131	18.90	3.93	23	<0.001***	20.48	3.64	1237	19.03	3.96	0.08
		[7,28]			[11,28]			[7,28]				[14,28]			[7,28]		
BAS score (Behaviour activation system)	1260	38.29	5.75	129	37.89	5.64	1131	38.33	5.77	23	<0.001***	37.43	6.10	1237	38.30	5.75	0.47
		[20,52]			[21,52]			[20,52]				[21,46]			[20,52]		
Delivery week											0.03						0.19
	1082	38.99	1.25	118	38.75	1.10	964	39.01	1.27	22		38.64	0.95	1060	38.99	1.26	
		[29,42]			[36,41]			[29,42]				[37,40]			[29,42]		
	**Total**				**Moderate child maltreatment**							**Severe child maltreatment**					
	**Missing**	**Number**	**%**	**Missing**	**Number**	**%**	**Missing**	**Number**	**%**	**Missing**	**p value**	**Number**	**%**	**Missing**	**Number**	**%**	**P value**
Partner (+)											0.81						0.01*
	4	1256	100.00	0	129	100.00	4	1127	100.00	0		23	100.00	4	1234	100.00	
Yes		1254	99.84		129	100.00		1125	89.29			23	100.00		1231	99.76	
No		2	0.16		0	0.00		2	0.16			0	0.00		2	0.16	
Employment											<0.01**						0.03*
	0	1260	100.00	0	94	100.00	0	1131	100.00	0		23	100.00	0	1237	100.00	
Full-time		521	41.35		35	27.13		486	42.97			5	21.74		516	41.71	
Part-time		153	12.14		16	12.40		137	12.11			1	4.35		152	12.29	
Temporary		71	5.63		5	3.88		66	5.84			0	0.00		71	5.74	
Homemaker		515	40.87		73	56.59		442	39.08			17	73.91		498	40.26	
Educational level											0.5						<0.01**
	0	1260	100.00	0	129	100.00	0	1131	100.00	0		23	100.00	0	1237	100.00	
Graduate degree		100	7.94		8	6.20		92	8.13			1	4.35		99	8.00	
University degree		664	52.70		64	49.61		600	53.05			9	39.13		655	52.95	
Junior or technical college		369	29.29		41	31.78		328	29.00			9	39.13		360	29.10	
High school		118	9.37		14	10.85		104	9.20			2	8.70		116	9.38	
Junior high school		9	0.71		2	1.55		7	0.62			2	8.70		7	0.57	
Annual household income											0.39						0.12
	10	1250	100.00	1	128	100.00	9	1122	100.00	0		23	100.00	10	1227	100.00	
<2 million yen		17	1.36		1	0.78		16	1.43			0	0.00		17	1.39	
2–4.9 million yen		250	20.00		33	25.78		217	19.34			8	34.78		242	19.72	
5–9.9 million yen		582	46.56		60	46.88		522	46.52			13	56.52		569	46.37	
>10 million yen		401	32.08		34	26.56		367	32.71			2	8.70		399	32.52	
Plurality											0.38					0.00	0.78
	174	1086	100.00	11	118	100.00	163	968	100.00	1		22	100.00	173	1064	100.00	
Singleton		1074	98.90		116	98.31		958	98.97			22	100.00		1052	98.87	
Twin		12	1.10		2	1.69		10	1.03			0	0.00		12	1.13	
Triplet		0	0.00		0	0.00		0	0.00			0	0.00		0	0.00	
Numbers of delivery											<0.001***		0.00			0.00	<0.001***
	178	1082	100.00	13	116	100.00	165	966	100.00	2		21	100.00	176	1061	100.00	
1		604	55.82		16	13.79	588	588	60.87			2	9.52		602	56.74	
2		381	35.21		72	62.07	309	309	31.99			9	42.86		372	35.06	
3		88	8.13		24	20.69	64	64	6.63			8	38.10		80	7.54	
4		7	0.65		3	2.59	4	4	0.41			1	4.76		6	0.57	
5 or more		2	0.18		1	0.86	1	1	0.10			1	4.76		1	0.09	
Psychiatric illness history (+)											0.28					0.00	0.42
(Missing value: n = 0)	0	1260	100.00	0	129	100.00	0	1131	100.00	0		23	100.00	0	1237	100.00	
Yes		161	12.78		19	14.73		142	12.56			2	8.70		150	12.13	
No		1099	87.22		110	85.27		989	87.44			21	91.30		1078	87.15	
Type of pregnancy											<0.01**						0.21
	12	1248	100.00	0	129	100.00	12	1119	100.00	0		23	100.00	12	1225	100.00	
Natural insemination		895	71.71		112	86.82		783	69.97			22	95.65		873	71.27	
Guidance of preferable timing of fertilization		139	11.14		7	5.43		132	11.80			0	0.00		139	11.35	
Artificial insemination		44	3.53		1	0.78		43	3.84			0	0.00		44	3.59	
Extrauteral insemination		82	6.57		4	3.10		78	6.97			0	0.00		82	6.69	
Microinsemination		87	6.97		5	3.88		82	7.33			1	4.35		86	7.02	
Others		1	0.08		0	0.00		1	0.09			0	0.00		1	0.08	
Method of birth											0.01*						0.53
	175	1260	100.00	11	118	100.00	164	967	100.00	1		22	100.00	174	1063	100.00	
Spontaneous vaginal birth		882	70.00		98	83.05		784	81.08			20	90.91		862	81.09	
Planned Caesarean section		109	8.65		16	13.56		93	9.62			2	9.09		107	10.07	
Emergency Caesarean section		87	6.90		2	1.69		85	8.79			0	0.00		87	8.18	
Others		7	0.56		2	1.69		5	0.52			0	0.00		7	0.66	

Missing indicates number of the participants with missing values. Self-administered short version of the PARS score indicates the total score of the self-administered short version of the Pervasive Developmental Disorders Autism Society Japan Rating Scale. ASD indicates autism spectrum disorder. ADHD indicates attention-deficit hyperactivity disorder. ASRS score indicates total score of the Adult ADHD Self Report Scale. BIS indicates behaviour inhibition system. BAS indicates behaviour activation system. BIS score indicates BIS subscale score of the Japanese version of the BIS/BAS Scale. BAS score indicates BAS subscale score of the Japanese version of the BIS/BAS Scale. “Moderate child maltreatment” and “Severe child maltreatment” refer to mothers at high risk of “moderate child maltreatment” and “severe child maltreatment”, as determined by the Child Maltreatment Scale (CMS). “At high risk” for “Moderate child maltreatment” indicates the CMS score was 3 or more. “Not at high risk” for “Moderate child maltreatment” indicates he CMS score 2 or less. “At high risk” for “Severe child maltreatment” indicates the CMS score was 7 or more. “Not at high risk” for “Severe child maltreatment” indicates the CMS score was 6 or less. “p value” indicates the p value of the t test or the chi-squared test for each variable between the “at high risk” or “not at high risk” group for child maltreatment tendency. “P value” indicates the p value of the t test or the chi-squared test for each variable between the “at high risk” or “not at high risk” group for child maltreatment. *, **, and ***indicate statistical significance in the analysis: p < 0.05, p < 0.01 and p < 0.001, respectively.

**Table 2:** Distribution of each scored item for moderate and severe child maltreatment and total score.

	Total (n = 1260)						At high risk of moderate child maltreatment (n = 129)						At high risk of severe child maltreatment (n = 23)					
	Not at all (score: 0)		Rarely (score: 1)		Sometimes (score: 2)		Not at all (score: 0)		Rarely (score: 1)		Sometimes (score: 2)		Not at all (score: 0)		Rarely (score: 1)		Sometimes (score: 2)	
	N	%	N	%	N	%	N	%	N	%	N	%	N	%	N	%	N	%
1. Leave the child crying	440	34.92	729	0.58	91	7.22	8	6.20	73	56.59	48	37.21	1	4.35	14	60.87	8	34.78
2. Don’t feed the child	1257	99.76	3	0.00	0	0.00	126	97.67	3	2.33	0	0.00	21	91.30	2	8.70	0	0.00
3. Don’t bathe them or change their underwear	1222	96.98	36	0.03	0	0.00	109	84.50	18	13.95	2	1.55	16	69.57	6	26.09	1	4.35
4. Yell at the child	1094	86.83	132	0.10	34	2.70	28	21.71	67	51.94	34	26.36	1	4.35	7	30.43	15	65.22
5. Spank the child	1201	95.32	55	0.04	4	0.32	74	57.36	51	39.53	4	3.10	4	17.39	16	69.57	3	13.04
6. Hit the child’s hand	1207	95.79	50	0.04	3	0.24	78	60.47	48	37.21	3	2.33	6	26.09	14	60.87	3	13.04
7. Hit the child’s head	1206	95.71	50	0.04	4	0.32	77	59.69	48	37.21	4	3.10	3	13.04	16	69.57	4	17.39
8. Slap the child’s face	1242	98.57	16	0.01	2	0.16	111	86.05	16	12.40	2	1.55	11	47.83	10	43.48	2	8.70
9. Pinch the child	1250	99.21	9	0.01	1	0.08	121	93.80	7	5.43	1	0.78	20	86.96	2	8.70	1	4.35
10. Hit the child with something	1257	99.76	2	0.00	1	0.08	126	97.67	2	1.55	1	0.78	20	86.96	2	8.70	1	4.35
11. Throw things at the child	1248	99.05	11	0.01	1	0.08	117	90.70	11	8.53	1	0.78	17	73.91	5	21.74	1	4.35
12. Cut the child’s hair (as a punishment or for fun)	1258	99.84	1	0.00	1	0.08	128	99.22	0	0.00	1	0.78	22	95.65	0	0.00	1	4.35
13. Confine the child to a closet	1253	99.44	6	0.00	1	0.08	122	94.57	6	4.65	1	0.78	19	82.61	3	13.04	1	4.35
14. Shut the child outside (balcony)	1251	99.29	8	0.01	1	0.08	121	93.80	7	5.43	1	0.78	18	78.26	4	17.39	1	4.35
15. Leave the child alone in the house	1188	94.29	68	0.05	4	0.32	89	68.99	36	27.91	4	3.10	12	52.17	8	34.78	3	13.04
16. Leave the child naked	1252	99.37	6	0.00	2	0.16	124	96.12	3	2.33	2	1.55	21	91.30	0	0.00	2	8.70
17. Leave the child alone in the car	1238	98.25	20	0.02	2	0.16	113	87.60	14	10.85	2	1.55	16	69.57	5	21.74	2	8.70
Total score: Mean (Standard deviation)	1.20 (1.68)						4.90 (2.92)						9.22 (4.55)					

“At high risk of moderate child maltreatment” indicates mothers with the Child Maltreatment Scale (CMS) score of 3 or more. “At high risk of severe child maltreatment” indicates mothers with the CMS score of 7 or more.

**Table 3:** The cumulative ratio of the total score of the Child Maltreatment Scale.

Total score	Number	%	Cumulative %
0	404	32.06	32.06
1	576	45.71	77.78
2	151	11.98	89.76
3	45	3.57	93.33
4	33	2.62	95.95
5	15	1.19	97.14
6	13	1.03	98.17
7	10	0.79	98.97
8	3	0.24	99.21
9	4	0.32	99.52
10	2	0.16	99.68
11	3	0.24	99.92
29	1	0.08	100.00
Total	1260	100.00	

“Cumulative %” indicates the cumulative ratio of the total score of the Child Maltreatment Scale.

**Table 4:** Coefficiency statistics of predictive factors used for multivariate analysis.

Variables	Tolerance	VIF
Psychiatric treatment history	0.948	1.054
Educational level	0.972	1.029
ASD traits (self-administered short version of the PARS score)	0.802	1.247
ADHD traits (ASRS score)	0.816	1.225
Behaviour inhibition system (BIS score)	0.798	1.252
Behaviour activation system (BAS score)	0.989	1.011

Coefficiency statistics indicates the results of the multicollinearity test in the linear regression. Tolerance and VIF indicates tolerance value and variance inflation factor in the multicollinearity test, respectively. ASD, self-administered short version of the PARS score, ADHD, ASRS score, BIS, BAS, BIS score, and BAS score: see Table 1’s legend.

### Cut-off scores of the Child Maltreatment Scale

Regarding the CMS cut-off score for “at high risk of moderate child maltreatment”, we considered the total score of each item. If the cut-off score was set at 1/2 (i.e. a mother is regarded as in high risk group of moderate child maltreatment if she has the score 2 or more), 2/3 (i.e. a mother is regarded as in high risk group if she has the score 3 or more and as not in high risk group if 2 or less), and 3/4 (i.e. a mother is regarded as in high risk group if she has the score 4 or more and as not in high risk group if 3 or less) with the total score, the ratio of the mothers at high risk of moderate child maltreatment in this study were 22.22%, 10.24%, and 6.67%, respectively. We also referred an Japanese epidemiological study performed in Osaka, the second largest city in Japan^16^. They reported 9.7% (3,320/34,341)^16^ of pregnant and puerperal women with psycho-social problems related to child maltreatment. We also checked the rationale for the cut-off score of “at high risk of moderate child maltreatment” from the clinical perspective concerning each item. “Item 1: leaving the child crying” can occur in mothers not exhibiting child-maltreatment behaviour (e.g., when mothers are tired). Due to such situations, it may be normal when this item’s score is coded as 2. However, in situations in which the score of the other items besides Item 1 was 1 or 2, this may imply that a baby’s health or safety may be at high risk and thus cannot be overlooked concerning childcare and prevention of child maltreatment. If the total score was 3 or more, the mothers were considered at least “positive” for items 2–17 irrespective of a positive or negative score for item 1. Thus, the CMS cut-off score of 2/3 for “at high risk of moderate child maltreatment” was regarded as valid and was used for Analysis 2. On the other hand, an epidemiological study^16^ also reported that pregnant and puerperal women at high risk of severe child maltreatment was 1.2% (470/38,204). Regarding the CMS cut-off score for “at high risk of severe child maltreatment”, we also considered the total score for each item. If it was set at 5/6, 6/7, and 7/8, the mothers at high risk of severe child maltreatment were 2.86%, 1.87%, and 1.03%, respectively. According to these data, the CMS cut-off score for “at high risk of severe child maltreatment” at 6/7 was regarded as appropriate and used for Analysis 3.

### Main analysis

#### Analysis 1: Linear regression analyses comparing four models, unadjusted and models 1–3

Table 5 shows the coefficients of the short version of the Pervasive Developmental Disorders (PDDs) Autism Society Japan Rating Scale (PARS)^17^ via self-administration, the short-form of the Adult ADHD Self-Report Scale (ASRS) scores^18^, and the subscale scores of the Japanese version of the BIS/BAS Scales^14,19^ for the CMS score using linear regression analysis. In the unadjusted model, one unit increase of the self-administered short version of the PARS score (coefficient (b) = 0.081, standard error of b (SE) = 0.016, and p < 0.001), ASRS score (b = 0.243, SE = 0.037, and p < 0.001), and BIS score (b = 0.057, SE = 0.012, p < 0.001) showed a significant increase in the CMS score. In Model 1 (adjusted for history of maternal psychiatric treatment and educational level) and Model 2 (adjusted for simultaneous ASD and ADHD traits in addition to adjustments in Model 1), the scores for the self-administered short version of the PARS (Model 1: b = 0.083, SE = 0.016, and p < 0.001; Model 2: b = 0.058, SE = 0.017, and p = 0.001) and ASRS (Model 1: b = 0.242, SE = 0.037, and p < 0.001; Model 2: b = 0.199, SE = 0.040, and p < 0.001) were also significantly associated with the CMS score. The BIS data showed a significant association with the CMS score in Model 1 (b = 0.060, SE = 0.012, and p < 0.001). In Model 3 (adjusted for simultaneous impulsivity [BIS and BAS] in addition to adjustments in Model 2), the BIS score remained significantly associated with the CMS score as well as the self-administered short version of the PARS and ASRS scores (BIS score: b = 0.031, SE = 0.013, and p = 0.018; the self-administered short version of the PARS score: b = 0.052, SE = 0.018, and p = 0.004; ASRS score: b = 0.178, SE = 0.041, and p < 0.001).

**Table 5:** Coefficients of ASD and ADHD traits and impulsivity for the Child Maltreatment Scale score.

		Unadjusted			Model 1			Model 2			Model 3		
		b	SE	p	b	SE	p	b	SE	p	b	SE	p
ASD traits	Self-administered short version of the PARS score (unit: 1 score)	0.081	0.016	<0.001***	0.083	0.016	<0.001***	0.058	0.017	0.001**	0.052	0.018	0.004**
ADHD traits	ASRS score (unit: 1 score)	0.243	0.037	<0.001***	0.242	0.037	<0.001***	0.199	0.04	<0.001***	0.178	0.041	<0.001***
Behaviour inhibition system	BIS score (unit: 1 score)	0.057	0.012	<0.001***	0.06	0.012	<0.001***	N/A			0.031	0.013	0.018*
Behaviour activation system	BAS score (unit: 1 score)	−0.006	0.008	0.474	−0.005	0.008	0.547	N/A			−0.011	0.008	0.175

ASD, self-administered short version of the PARS score, ADHD, ASRS score, BIS score, and BAS score: see Table 1’s legend. Model 1 adjusted for maternal psychiatric treatment history and educational level. Model 2 adjusted ASD and ADHD traits simultaneously in addition to Model 1. Model 3 adjusted impulsivity (BIS and BAS) simultaneously in addition to Model 2. *, **, and ***indicates statistical significance in the analysis: p < 0.05, p < 0.01 and p < 0.001, respectively.

### Sub-analyses

#### Analysis 2: Logistic regression analysis of Model 3 using the cut-off score for “at high risk of moderate child maltreatment” as the dependent variable

The results of the multivariate analysis in Analysis 2 are shown in Table 6. The BIS score (p = 0.016 and AOR = 1.068 [95% CI = 1.012–1.126]) as well as the self-administered short version of the PARS score (p = 0.014 and AOR = 1.083 [95% CI = 1.016–1.153]) showed statistically significance. The area under the curve (AUC), sensitivity, and specificity were 0.645, 0.512 and 0.755, respectively.

**Table 6:** Multivariate analyses for “at high risk of moderate child maltreatment” and “at high risk of severe child maltreatment”.

Variable	At high risk of moderate child maltreatment Analysis 2 (n = 1,260)			At high risk of severe child maltreatment Analysis 3 (n = 1,260)		
	p value	AOR	95%CI	p value	AOR	95%CI
Psychiatric treatment history	0.571	0.855	0.497–1.472	0.205	0.374	0.082–1.711
Educational level	0.474	0.958	0.953–1.077	0.073	0.797	0.621–1.021
ASD traits (self-administered short version of the PARS score)	0.014*	1.083	1.016–1.153	0.168	1.098	0.961–1.254
ADHD traits (ASRS score)	0.117	1.128	0.970–1.311	0.022*	1.437	1.054–1.959
Behaviour inhibition system (BIS score)	0.016*	1.068	1.012–1.126	0.585	1.034	0.917–1.167
Behaviour activation system (BAS score)	0.205	0.979	0.948–1.012	0.327	0.964	0.895–1.038

ASD, self-administered short version of the PARS, ADHD, ASRS score, BIS score, and BAS score: see Table 1’s legend. p value, AOR, and 95% CI indicates the values of p values, adjusted odds ratios, and 95% confidence intervals of the odd ratios in the logistic regression analysis, respectively. *Indicates statistical significance and marginal significance in the analysis (p < 0.05), respectively. Analysis 2: A multivariable logistic regression using a model with a binary variable “at high risk of moderate child maltreatment” as a dependent variable and maternal psychiatric treatment history, educational level, ASD trait, ADHD trait, BIS, and BAS as independent variables. Analysis 3: A multivariable logistic regression using a model with a binary variable “at high risk of severe child maltreatment” as a dependent variable and maternal psychiatric treatment history, educational level, ASD trait, ADHD trait, BIS, and BAS as independent variables.

#### Analysis 3: Logistic regression analysis of Model 3 using the cut-off score for “at high risk of severe child maltreatment” as the dependent variable

The results of the multivariate analysis in Analysis 3 are shown in Table 6. The ASRS score (p = 0.022 and AOR = 1.437 [95% CI = 1.054–1.959]) showed statistically significance. The AUC, sensitivity, and specificity were 0.762, 0.696 and 0.781, respectively.

## Discussion

### Principal findings

This study tested the hypothesis of impulse control disability, as outlined in the BIS/BAS model, and developmental disorder traits being associated with child maltreatment. Three new findings were revealed. First, we demonstrated that excessive inhibition of behaviour and affect, thus, impulse control disability, is significantly associated with child maltreatment in addition to maternal developmental disorder traits. Second, ADHD traits were significantly associated with child maltreatment, even when ASD traits were considered. Third, ASD and ADHD traits may differentially affect the severity of child maltreatment. ASD traits and poor impulse control with excessive inhibition of behaviour and affect were shown to be important risk factors for moderate child maltreatment. ADHD traits were revealed not to be a risk factor for being in high risk group of child maltreatment tendency but high risk group of child maltreatment, with higher risk of child maltreatment.

### Strengths and weaknesses of this study

To our knowledge, this is the first report that demonstrated the importance of BIS as a risk factor for child maltreatment. We performed this study in a highly-populated area of Tokyo. The women enrolled in this study were from diverse backgrounds regarding socioeconomic status. Thus, our results are representative and holistically present evidence of risk factors for child maltreatment.

However, this study has several limitations. First, we assessed child maltreatment using a self-reporting questionnaire rather than obtaining information from hospitals or child protection services. Hence, we could not confirm actual child maltreatments of the mothers. However, since self-reporting questionnaires are the primary source for estimating the prevalence of child maltreatment and have also been used to estimate the incidence of maltreatment^20,21^, we consider our results to be valid. The self-administered short version of the PARS, ASRS, and BIS/BAS Scales are also self-reporting questionnaires and may include measurement bias regarding self-recognition. Second, the study sample may not represent the overall Japanese population. The Setagaya Ward, where the study was conducted, is a residential area in metropolitan Tokyo. Some differences in the psychosocial characteristics analysed in this study may exist between the metropolitan and rural areas of Japan. Third, we tested our hypothesis using a previous study^1^’s model that investigated the association of developmental disorder traits with child maltreatment across the spectra of maternal psychiatric treatment history and educational level. While our results suggested that these models may predict child maltreatment to some extent, there are other maternal risk factors for child maltreatment^22–24^ not considered in this study.

### Comparison with other studies

To note, there was a discrepancy with the previous study^1^ in terms of the association between ADHD traits and child maltreatment. In that study, the association was not significant when ASD traits were adjusted. In our study, however, the association remained significant when ASD traits were adjusted in Analysis 1. The previous study was conducted at one national children’s hospital, which might have resulted in substantial differences in patient cohorts, given that the 25, 50, and 75^th^ percentile values of the ASRS total scores in that study were 0, 0, and 1 ([Min, Max] = [0, 5]) compared with 0, 1, and 2 ([Min, Max] = [0, 6]) in the present study, respectively. We postulated that the patient population in the previous study may have included a smaller number of women with ADHD than our cohort, which would have weakened the effect of ADHD traits in the association analyses. This may be the reason why that study did not show a significant association between ADHD and child maltreatment when ASD was adjusted. Our results were consistent with the results of the logistic regression analysis, which indicated that ADHD traits are an important risk factor for more severe child maltreatment cases compared with ASD traits. We found that ASD traits were important risk factors, consistent with the previous study^1^. The current study considered impulse control using the same linear regression models^1^ in which revealed an association with developmental disorder traits and child abuse.

The significant association between high BIS levels and child maltreatment suggested the importance of paying close attention to maternal characteristics related to excessively active BIS, as these mothers may be prone to child maltreatment. According to Gray’s theory, impulse control disability with high BIS levels leads to anxiety and, consequently, a shift toward the direction of avoidance^12^. This excessive inhibition of behaviour and affect may lead to immense anxiety concerning parenting and avoidance of childcare. Previous studies have described an association between maternal anxiety and child maltreatment^25,26^. It is critically important for relevant healthcare professionals to understand maternal anxieties about childcare concerning mothers with high BIS levels who are at risk of child maltreatment. Interventions to relieve maternal anxieties and to empower mothers with parenting skills to shift them from avoidance behaviour to positive attitudes towards childcare may be effective in provision of support and preventing child maltreatment.

Our results shed light on the necessity for conducting psychosocial risk assessments on pregnant women to detect impulse control disability and developmental disorder traits, which can, in turn, be useful in the context of child maltreatment prevention. In Japan, there is a form, provided by the local government, aimed at supporting mothers at high risk for psychosocial problems in order to prevent child abuse. Within this form, maternal mental health problems and other health problems are assessed. These problems include psychiatric diseases, mental retardation, anxiety, chronic diseases, and physical disability^16,27^. However, this form does not include any item pertaining to the assessment of impulsivity. Fujiwara *et al*. investigated the association between psychosocial risk factors and child abuse at 4 months postpartum^28^. The multivariate analysis included the following: mother’s age, gestational weeks when turning in a pregnancy notification form to the local government, parity, unwanted pregnancy, support from the baby’s grandmother after delivery, support from others after delivery, worries about pregnancy or delivery, and depression. Their work revealed that young age, primipara, and unwanted pregnancies predicted child abuse at 4 months postpartum. However, their predictive factors did not include maternal psychosocial factors related to personality. Further research is needed to develop a convenient and highly sensitive assessment tool that detects and/or evaluates maternal impulsivity and developmental disorder traits during pregnancy and postpartum periods.

### Implications for clinicians and policymakers

As aforementioned, we revealed impulsivity control disability as an important risk factor for child maltreatment. Psychotherapeutics such as insight-oriented psychotherapy, cognitive behaviour therapy, contingency management, and pharmacological approaches, which are evidence-based, may be beneficial for mothers prone to child maltreatment and impulse control disorder^29^. In addition, this study demonstrated the importance of both ASD and ADHD traits as risk factors for child maltreatment. Individuals with ASD exhibit characteristics such as persistency, repetitive behaviours, and disabilities with social communication and social interaction across multiple contexts^2^. Similarly, individuals with ADHD had characteristics such as hyperactivity and inattentions^2^. Several effective therapies for individuals with ASD and ADHD have been developed^30,31^. Our results suggested that a therapeutic approach targeting developmental disorder characteristics could be beneficial for abusive mothers with ASD or ADHD traits. Additionally, assessments of developmental disorder traits and impulse control disorder for abusive mothers may help elucidate the difficulties they are facing, which may in turn lead to the creation of better support programs for them.

### Unanswered questions and future research

Child maltreatment results from not only maternal characteristics such as maternal impulse control disability and developmental disorder traits but also multiple causes associated with other psychosocial factors^32,33^. Based on our results, further research is warranted to investigate these factors. Impulsivity was revealed as a vulnerability marker for substance-use disorders^34^, such as addiction^35,36^. Child abuse has been described as “not a psychiatric disorder” but as “other conditions that may be a focus of clinical attention” in the Diagnostic Statistical Manual of Mental Disorders Fifth Edition (DSM-5)^2^. Our results suggested the possibility of child abuse being related to impulsivity control disorder. At present, there is a category called “Disruptive, Impulse-Control, and Conduct Disorders” in the DSM-5. We propose that child abuse to be considered as one of its sub-categories. Child abuse often results in deep psychological scarring in children. However, psychiatric therapeutic approaches are needed not only for the children but also for the abusive mothers. Further research is needed to examine child abuse from the perspective of impulsivity control disorder.

## Methods

### Study design

This was a longitudinal study that began in September 2012. Participants were recruited between December 2012 and May 2013 (Figure 1). Written informed consent was obtained from all participants. We performed surveys at five time points: 20 weeks gestation and the first few days, two weeks, one month, two months, and three months after delivery. The participants were given subsequent questionnaires unless they either withdrew or did not respond to the preceding questionnaire. The data collected at T1 (20 weeks gestation) and T2 (the first few days after delivery) were paper-based self-administered questionnaires or an iPad (Apple, Inc.) questionnaire application (MMONGA; Xware Corp., Tokyo, Japan). Two months postnatal (T3) and three months postnatal (T4) questionnaires were sent to the participants and returned via mail.

### Ethics approval

This study was approved by the research ethics committee of the National Centre for Child Health and Development in Tokyo, Japan and carried out in accordance with established, institutional ethical standards.

### Participants

Participants included in this study were pregnant women (20 weeks gestation) who were scheduled for delivery in any of the 14 obstetrics hospitals in the Setagaya Ward. Setagaya Ward is located in the urban area of Tokyo, and its population was 860,935 on December 1, 2012^37^. The number of live births, total fertility rate, and the birth rate per 1,000 persons were 7731, 0.98, and 9.18, respectively, in 2013^38^. All hospitals with obstetrics wards in Setagaya were involved in this study. Participants who planned to deliver at a hospital outside of Setagaya were excluded. If a participant gave birth to a stillborn, the hospital informed our research team, and that participant was excluded from the study. Mothers with stillborn babies were cared for by the obstetricians, midwives, and nurses in the same, standard routine of clinical care. If a participant had psychiatric problems, she received care by the perinatal staff and was referred to other psychiatric clinics or hospitals according to normal protocol.

### Measures

#### Assessment of ASD traits

We used the short version of the PARS^17^, conducted via self-administration, in T1 to assess the mothers’ ASD traits. The original version, the Pervasive Developmental Disorders Autism Society Japan Rating Scale – Text Revision short version, is interview-based^39,40^ and has good reliability (α = 0.83) and validity (Pearson’s correlation of its full version with Autism Diagnostic Interview, Revised^41^ = 0.41)^17^. The short version of the PARS (interview-based) consists of 12 items^17^. These 12 items are based on the main symptoms of ASD as listed in the Diagnostic Statistical Manual of Mental Disorders Fourth Edition Text Revision (DSM-IV-TR)^42^, i.e., qualitative impairment in social interaction and communication and restricted repetitive and stereotyped patterns of behaviour, interests, and activities. In this study, we used this version via self-administration as earlier noted, which has previously been used to investigate the association between developmental disorder traits and child maltreatment^1^. While the Autism Spectrum Quotient (AQ)^43^ is known for its ability to detect ASD traits of adults, previous studies revealed that the self-administered short version of the PARS has higher association with child maltreatment than the AQ^44–46^. Therefore, we used the self-administered short version of the PARS score as a continuous variable.

#### Assessments of ADHD traits

T3 data involved a questionnaire on ADHD traits. ADHD traits were measured by the short-form of the Adult ADHD Self-Report Scale (ASRS)^18^. It consists of six questions covering attention deficit and hyperactivities based on DSM-IV-TR with valid sensitivity and specificity (68.7% and 99.5%, respectively). The ASRS score was used in the analyses as a continuous variable.

#### Assessments of maternal impulse control

The behavioural inhibition/behavioural activation scales (BIS/BAS Scales) were used to assess maternal impulse control in T3. The BIS/BAS Scales, which can measure impulsivity, were developed by Craver and White^14^ on the basis of Gray’s personality theory^47,48^. They tested the validity of the BIS/BAS Scales, in which, the impulsive quality measured by the Disinhibition-Constraint scale^49^ was correlated^14^. Its Japanese version has been validated^19^ (See Supplementary Information S1). A greater BIS score reflects a greater prone to anxiety, provided the person is exposed to proper situational cues such as punishment, non-reward, and novelty^50^. Greater BAS scores have also been reported to relate to psychopathy^5,51^.

#### Assessments of child maltreatment

The T4 data used in this study were based solely on the child abuse and neglect questionnaire. Hence, we used the CMS that was developed in Japan^15^ (See Supplementary Information S2). It is composed of 17 items; for each item, 0 (not at all), 1 (rarely), or 2 (sometimes) points are marked, and each point is summed for total score. Its validity has been previously demonstrated (α = 0.77) in an urban community in Japan^15^.

In Japan, most local administrative governments have two types of centres that manage child maltreatment. Child and family support centres provide advice and counselling for families and children for moderate child maltreatment cases, while child protection centres manage severe maltreatment cases to protect children. Thus, the present study classified child maltreatment into the two categories of moderate and severe child maltreatment. We determined the appropriate CMS cut-off scores for moderate and severe child maltreatment by referencing the distribution of our results and those of a previous Japanese epidemiological study^16^, with clinical considerations based on those data. Via our results, we set the cut-off scores of “at high risk of moderate child maltreatment” and “at high risk of severe child maltreatment” at 2/3 (i.e. a mother is regarded as in high risk group if she has the score 3 or more and as not in high risk group if 2 or less) and 6/7 (i.e. a mother is regarded as in high risk group if she has the score 7 or more and as not in high risk group if 6 or less), respectively.

#### Assessment of demographic, clinical, and socioeconomic characteristics

Demographic, clinical, and socioeconomic data (Table 1) were also collected at T1 (partner existence, employment, household income, history of psychiatric treatment, educational level, type of pregnancy, and fertilization) and T2 (age, delivery week, plurality, numbers of delivery, and method of birth).

### Data preparation

An electronic database was developed using the collected data. All data input into the database were double-checked. All measurement ranges, means, standard deviations, distributions, outliers, and logical errors were examined.

### Privacy protection

All information that could identify individual participants was not input into the database with the exception of participants’ identification numbers.

### Statistical analyses

#### Main analysis

Analysis 1: linear regression analyses comparing four models

To investigate the association of maternal developmental disorder traits and impulse control with child maltreatment, linear regression analyses were performed based on the same models outlined in a previous study^1^. We analysed four models (the unadjusted model and Models 1–3): the unadjusted model; Model 1 was adjusted by a history of psychiatric treatment and educational level (i.e., Y [child maltreatment] = β0 + β1*[psychiatric treatment history] + β2*[educational level] + β3*[ASD trait or ADHD trait or BIS score or BAS score]; Model 2 was simultaneously adjusted by ASD and ADHD traits in addition to adjustments detailed in Model 1 (i.e., Y [child maltreatment] = β0 + β1*[psychiatric treatment history] + β2*[educational level] + β3*[ASD trait] + β4*[ADHD trait]); and Model 3 was simultaneously adjusted by ASD and ADHD traits and BIS/BAS in addition to the adjustments of Model 1 (i.e., Y [child maltreatment] = β0 + β1*[psychiatric treatment history] + β2*[educational level] + β3*[ASD trait] + β4*[ADHD trait] + β5*[BIS score] + β6*[BAS score]). The adjustment variables of Model 1 were determined via a history of psychiatric treatment theoretically associated with developmental traits and child maltreatment^52–55^ as per a previous study^1^. Multicollinearities of the linear regression models were estimated. The presence of multicollinearity was judged with a tolerance value and VIF (less than 0.4 and greater than 2.5, respectively)^56^. To investigate the validity of Model 3 for predicting moderate child maltreatment and severe child maltreatment, we performed two types of logistic regression analyses: Analyses 2 and 3.

### Sub-analyses

Analysis 2: logistic regression analysis of Model 3 using the cut-off score for “at high risk of moderate child maltreatment” as the dependent variable

We used logistic regression analysis to determine if maternal impulsivity (BIS/BAS) was associated with moderate child maltreatment, adjusting for maternal psychiatric treatment history and educational level, ASD traits, and ADHD traits. The participants were classified into two groups, “at high risk of moderate child maltreatment” and “not at high risk of moderate child maltreatment,” by the cut-off score for “at risk of moderate child maltreatment” (2/3). A logistic regression was performed with the two groups as the dependent variables and with the same independent variables as that of Model 3 (i.e., maternal psychiatric treatment history, educational level, ASD traits, ADHD traits, behaviour inhibition system, and behaviour activation system). The sensitivity and specificity of the model were evaluated using the Youden’s Index.

Analysis 3: logistic regression analysis of Model 3 using the cut-off score for “at high risk of severe child maltreatment” as the dependent variable

Analysis 3 was performed to investigate if maternal impulsivity (BIS/BAS) was associated with severe child maltreatment, adjusting for ‘current or past psychiatric treatment history,’ education level, ASD traits, and ADHD traits. The participants were classified into two groups, “at high risk of severe child maltreatment” and “not at high risk of severe child maltreatment,” by the cut-off score for severe child maltreatment (6/7). As per Analysis 2, Analysis 3 was performed using the two groups with the cut-off score for “at high risk of severe child maltreatment” as the dependent variable. The variables with p values of 0.05 or less were considered as statistically significant for all analyses. Data analyses were conducted using JMP version 11.2 for Windows (SAS Inc., Tokyo, Japan).

## Electronic supplementary material

Supplementary Information
