# Supplementary material for: Maternal impulse control disability and developmental disorder traits are risk factors for child maltreatment
Source: Sci Rep. 2017 Nov 14;7:15565. doi: 10.1038/s41598-017-14666-5 (PMC5686103; doi:10.1038/s41598-017-14666-5)
Supplement: Supplementary file 1 — Supplementary Information [file 41598_2017_14666_MOESM1_ESM.pdf]

Maternal impulse control disability and developmental disorder traits are risk factors for child maltreatment

Yoshiyuki Tachibana[1*], Kenji Takehara[2], Naoko Kakee[3], Masashi Mikami[4], Eisuke Inoue[4,¤1], Rintaro Mori[2], Erika Ota[2,¤2], Tomoe Koizumi[5], Makiko Okuyama[6], Takahiko Kubo[7,¤3]

1. Division of Infant and Toddler Mental Health, Department of Psychosocial Medicine, National Centre for Child Health and Development, Tokyo, Japan
2. Department of Health Policy, National Research Institute for Child Health and Development, Tokyo, Japan
3. Division of Bioethics, National Centre for Child Health and Development, Tokyo, Japan
4. Department of Biostatistics, Clinical Research Centre, National Centre for Child Health and Development, Tokyo, Japan
5. National Research Institute for Child Health and Development, Tokyo, Japan
6. Department of Psychosocial Medicine, National Centre for Child Health and Development, Tokyo, Japan
7. Department of Perinatal Medicine and Maternal Care, National Center for Child Health and Development, Tokyo, Japan

¤1 Current address: Division of Medical Informatics, St. Mariann University School of Medicine, Kawasaki, Japan

¤2 Current address: Global Health Nursing, Graduate School of Nursing Science, St. Luke's International University, Tokyo, Japan

¤3 Current address: Shirota Obstetrical and Gynecological Hospital, Zama, Japan

Correspondence to:
Yoshiyuki Tachibana, M.D., Ph.D
Division of Infant and Toddler Mental Health, Department of Psychosocial Medicine,
National Center for Child Health and Development
2-10-1 Okura, Setagaya-ku, Tokyo, Japan 157-8535
Tel/Fax: +81 (0) 3 3416 0181;
E-mail: tachibana-y@ncchd.go.jp

Supplementary Information

Supplementary Information S1. The Japanese version of the BIS/BAS Scales (English translation)

Each item of this questionnaire is a statement that a person may either agree with or disagree with. For each item, indicate how much you agree or disagree with what the item says. Please respond to all the items; do not leave any blank. Choose only one response to each statement. Please be as accurate and honest as you can be. Respond to each item as if it were the only item. That is, don't worry about being "consistent" in your responses. Choose from the following four response options:

1 = very true for me
2 = somewhat true for me
3 = somewhat false for me
4 = very false for me

1. Even if something bad is about to happen to me, I rarely experience fear or nervousness.
2. I go out of my way to get things I want.
3. When I'm doing well at something I love to keep at it.
4. I'm always willing to try something new if I think it will be fun.
5. When I get something I want, I feel excited and energized.
6. Criticism or scolding hurts me quite a bit.
7. When I want something I usually go all-out to get it.
8. I will often do things for no other reason than that they might be fun.
9. If I see a chance to get something I want I move on it right away.
10. I feel pretty worried or upset when I think or know somebody is angry at me.
11. When I see an opportunity for something I like I get excited right away.
12. I often act on the spur of the moment.
13. If I think something unpleasant is going to happen I usually get pretty "worked up."
14. When good things happen to me, it affects me strongly.
15. I feel worried when I think I have done poorly at something important.
16. I crave excitement and new sensations.
17. When I go after something I use a "no holds barred" approach.
18. I have very few fears compared to my friends.

19. It would excite me to win a contest.
20. I worry about making mistakes.

Items other than 1 and 18 are reverse-scored.

BIS: 1(R), 6, 10, 13, 15, 18(R), 20

BAS: 2, 3, 4, 5, 7, 8, 9, 11, 12, 14, 16, 17, 19

References:

1. Carver, C. S., & White, T. L. (1994). Behavioral inhibition, behavioral activation, and affective responses to impending reward and punishment: The BIS/BAS Scales. *Journal of Personality and Social Psychology*, 67, 319-333.
http://www.psy.miami.edu/faculty/ccarver/sclBISBAS.html

2. Takahashi, Y., Yamagata, S., Kijima, N., Shigemasu, K., Ono, Y., Ando, J. Gray's temperament model: development of Japaneseversion of BIS/BAS scales and a behavior genetic investigation using the twin method. *Journal of Personality* **15**, 276-289 (2007). (in Japanese)

Supplementary Information S2. The Child Maltreatment Scale (English translation)

Do you treat your child as below? Please respond to the each item.

0 = not at all
1 = rarely
2 = sometimes

1. Leave the child crying
2. Don't feed the child
3. Don't bathe them or change their underwear
4. Yell at the child
5. Spank the child
6. Hit the child's hand
7. Hit the child's head
8. Slap the child's face
9. Pinch the child
10. Hit the child with something
11. Throw things at the child
12. Cut the child's hair (as a punishment or for fun)
13. Confine the child to a closet
14. Shut the child outside (balcony)
15. Leave the child alone in the house
16. Leave the child naked
17. Leave the child alone in the car

Reference:

Tokunaga, M., Ohara M., Kayama, M., Yoshimura, K., Mitsuhashi, J., Senoo, E. A child maltreatment survey in the Tokyo metropolitan area. *Kosei no Shihyo* **47**, 3-10 (2000). (in Japanese)
